# Specific timely appointments for triage to reduce wait times in a medical outpatient clinic: protocol of a pre-post study with process evaluation

**DOI:** 10.1186/s12913-019-4660-6

**Published:** 2019-11-12

**Authors:** Annie K. Lewis, Nicholas F. Taylor, Patrick W. Carney, Katherine E. Harding

**Affiliations:** 1 Allied Health Clinical Research Office and Department of Neurosciences, Eastern Health, 5 Arnold St, Box Hill, Victoria 3128 Australia; 2 School of Allied Health, Health Services and Sport, La Trobe University, Kingsbury Drive, Bundoora, Victoria 3086 Australia; 3 Neurosciences, Monash University, 21 Chancellors Walk, Clayton, Victoria 3800 Australia; 4 The Florey Institute for Neuroscience and Mental Health, Melbourne Brain Centre, Burgundy Street, 3084 Heidelberg, Australia

**Keywords:** Outpatient clinics, Waiting lists, Access, Appointments and schedules, Patient flow

## Abstract

**Background:**

Managing demand for services is a problem in many areas of healthcare, including specialist medical outpatient clinics. Some of these clinics have long waiting lists with variation in access for referred people. A model of triage and appointment allocation has been developed and tested that has reduced waiting times by about a third in community outpatient services. This study aims to determine whether the model can be applied in the setting of a specialist medical outpatient clinic to reduce wait time from referral to first appointment.

**Methods:**

A pre-post study will collect data before and after implementing the Specific Timely Appointments for Triage (STAT) model of access and triage. The study will incorporate a pre-implementation period of 12 months, an implementation period of up to 6 months and a post STAT-implementation period of 6 months. The setting will be the epilepsy clinic at a metropolitan health service in Melbourne. Included will be all people referred to the clinic, or currently waiting, during the allocated periods of data collection (total sample estimated *n* = 975). Data routinely collected by the health service and qualitative data from staff will be analysed to determine the effects of introducing the STAT model. The primary outcome will be wait time, measured by number of patients on the wait list at monthly time points and the mean number of days waited from referral to first appointment. Secondary outcomes will include patient outcomes, such as admission to hospital while waiting, and service outcomes, including rate of discharge. Analysis of the primary outcome will include interrupted time series analysis and simple comparisons of the pre and post-implementation periods. Process evaluation will include investigation of the fidelity of the intervention, adaptations required and qualitative analysis of the experiences of clinic staff.

**Discussion:**

Prompt access to service and optimum patient flow is important for patients and service providers. Testing the STAT model in a specialist medical outpatient clinic will add to the evidence informing service providers and policy makers about how the active management of supply and demand in health care can influence wait times. The results from this study may be applicable to other specialist medical outpatient clinics, potentially improving access to care for many people.

## Background

Waiting for healthcare services is a problem across the care continuum [1] in emergency departments [2], acute hospitals [3] and in ambulatory, community and outpatient settings [4]. Much work has been done on patient flow particularly in the acute setting [5] but there has been increased attention focusing on access, triage and patient flow in non-bed-based services [6–8]. As chronic disease management relies on responsive outpatient services, demand and wait lists for these services has increased [8]. Specialist medical outpatient clinics can have very long wait lists, sometimes up to several years, and some lower priority patients may never receive an appointment [8]. These long waiting times have implications for patient safety with potential risk of deterioration, anxiety and avoidable attendances to the emergency department [8–11]. Waiting for anything within health services is considered to contribute to wastage and avoidable costs [12]. Given the scale and implications of delays in outpatient care, timely and equitable access to specialist medical outpatient clinics is a current government priority [13]. Growing demand and waitlists can appear overwhelming, but the problem should not be seen as intractable [14]. In fact it is a common observation in health services that many waiting lists are relatively stable over time, indicating that supply and demand are actually well balanced, but an entrenched backlog of waiting patients leads to constant delays in care [7, 15].

An innovative model of access and triage, which challenges assumptions about supply and demand, has been developed and evaluated in the community outpatient setting [6, 16, 17]. This setting refers to services provided external to the hospital and includes ambulatory care, community health, and community-based outpatient services. The model, called STAT (Specific Timely Appointments for Triage) [6] is based on data modelling that calculates how many new appointments per week are required to keep up with demand. It also requires a system which maintains patient flow is embedded into practice so that the service actively keeps up with referrals by protecting appointments for new patients in clinician schedules. When commencing the STAT model, a short term, one-off, targeted intervention is implemented that aims to eliminate or markedly reduce the wait list. Each patient referred can then be offered the next available appointment without being prioritised for urgency, as they will *all* be seen in a timely manner. Priority decisions are made at the initial appointment by the treating clinician regarding follow-up or review appointments. Maintaining a constant flow of new patients into the service creates a driver to maximise efficiency across all aspects of patient flow; for example, by modifying practices related to review appointments, discharges and other clinical and administrative processes.

The STAT model showed success in two pilot trials [16, 17] and was then tested in a stepped wedge cluster randomised controlled trial involving eight diverse community outpatient services (including continence clinics, paediatric and adult community health services, community rehabilitation and physiotherapy outpatient clinics) and over 3000 patients [6]. A 34% reduction in waiting time was attributable to the intervention, which was found to be cost effective, sustained at 12 months and well received by staff [18]. It is not known whether the model could be applied to reduce wait times in specialist medical outpatient clinics.

There are similarities between community outpatient services and specialist medical outpatient clinics in that both are attended by community-dwelling people for assessment who are often scheduled for review appointments. They rarely deal with time critical events, with resulting acceptance of waitlists as a way of managing demand. Both service types play an important role in assisting people to manage non-infectious chronic diseases. The efficient management of non-bed-based services impacts on the demand for acute medical care and patient flow throughout the healthcare continuum [19].

There are also some differences between specialist medical outpatient clinics and the community outpatient services in which the STAT model has been trialed to date. Specialist medical outpatient clinics are often medical-only, single-discipline clinics, and are part of the hospital system, along with emergency department and inpatients [19]. In Australia, the funding streams, and reporting, data and governance requirements are quite different, which impacts on the administration of the two service types. The scale of the problem is much larger in specialist medical outpatient clinics where some people have been waiting years for an appointment and the number waiting is significantly greater than the community outpatient services that participated in previous trials.

Delayed access to specialist medical outpatient clinics is a serious problem [20]. This project aims to determine whether an evidence-based model of access, STAT, that reduced waiting time in community outpatient services [6] can be successfully applied to a specialist medical outpatient clinic serving patients with epilepsy to achieve reduced wait time. The specific research questions are: [i] Does the STAT model reduce the average wait time for people referred to a medical specialist epilepsy clinic? [ii] Does the STAT model have an impact on patient and service outcomes? [iii] What can be learned about the process of implementing STAT in this setting?

## Methods

### Design and setting

**Fig. 1:**
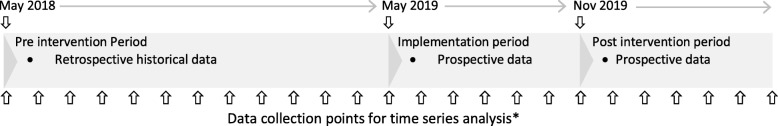
Data collection timeline. *Number of people on the waiting list to be collected monthly throughout implementation period and post intervention period (including at the conclusion of the final month), and as available during the pre-intervention period with some missing data anticipated. Average waiting time per month is expected to be available for all time points

This is a pre-post study with process evaluation. Routine service data will be collected for a 12-month period prior to the intervention (retrospective data) and for 6 months after the intervention (prospective data). A six-month period is planned for implementation, but the post-implementation period will commence sooner if the existing waiting list has been reduced to below a target threshold (Fig. 1).

A process evaluation will be completed towards the end of the post-implementation phase. It will be based on Medical Research Council’s guidance and framework, combining qualitative and quantitative data to provide information on context, implementation and mechanism of impact [21].

The setting is a first-seizure and general epilepsy clinic at a public metropolitan hospital, referred to as the ‘Epilepsy Clinic’. This specialist medical outpatient clinic runs for one afternoon each week and is staffed by four neurologists. People are referred to this clinic if they have had a first suspected seizure or have previously been diagnosed with epilepsy and require neurologist care. The clinic is funded by Medicare, the Australian universal healthcare system, with no out-of-pocket expense incurred by patients. Upon receipt of a referral, a neurologist triages and allocates each referral a priority code of 1 to 4, with 1 being the most urgent. In general, priority 1 and 2 patients are seen as per government guidelines, within 1 week and 1 month, respectively. However, those triaged as less urgent, priority 3 and 4 are placed on a waitlist and may wait for several years. Over the last 2 years there have been between 600 and 700 people waiting for appointments at any given time, suggesting that supply has been keeping up with demand, but no progress has been made into reducing the entrenched backlog of waiting patients or getting to the lowest priority patients. The longest waiters have been on the waiting list for over 7 years.

### Participants

The participants are people who have been referred to the epilepsy clinic and who received their first appointment during the 12 month period prior to implementation (pre-intervention cohort) or during the 6 months following implementation of the STAT model (post-implementation cohort). In addition, the number of patients on the waiting list (referred but not allocated an appointment) will be tracked monthly from the beginning of the implementation period to the end of the post-implementation period.

Clinic staff, including neurologists, administrative staff and management, will be invited to participate in a focus group where data will be collected for evaluation of process.

### Recruitment and consent

Low risk ethics approval has been granted by the Eastern Health and La Trobe University human research ethics committees (LR19/014). Individual patient consent will not be sought as the data for analysis is information routinely collected to which the clinical members of the research team would normally have access. Staff participating in the focus group will provide written informed consent.

## Intervention

The intervention is the implementation of the 5-step STAT model as described in the STAT handbook [22].

Step 1 requires data to be gathered about the demand for service over the preceding year as well as the ‘supply’ or clinic capacity. The essential demand data will be the number of people referred, the number of referrals that are rejected or withdrawn prior to being provided with an appointment, and the number of people who failed to attend.

Step 2 involves use of the data to calculate the number of new patient appointments required weekly to keep up with demand, including a buffer of 15% to allow for appointments that will be unavailable due to routine factors such as staff leave. These calculations also allow for patients who fail to attend and are rescheduled, using additional appointments.

Annual data are worked into an equation as follows:

$$ \left\{\raisebox{1ex}{$\left(x-y+z\ \right)$}\!\left/ \!\raisebox{-1ex}{$52$}\right.\right\}\times 1.15= Required\ number\ of\ appointment\ slots/ week $$

$$ x= number\ of\ referrals\ received\ \mathrm{annually} $$

$$ y= number\ of\ referrals\ rejected\ or\ withdrawn\ annually $$

$$ z= number\ of\ people\  who\  fail\ \mathrm{t}o\  attend\ and\  are\  provided\ with\ a\  new\  appointment $$

Step 3 is the reduction or elimination of the backlog of people currently waiting. Previous STAT trials have used different strategies to achieve this [22] and a multi-factor strategy will be developed in consultation with epilepsy clinic staff and key stakeholders. It will be emphasised that this is one-off, and once the waitlist is at the target level, the STAT model is designed to maintain patient flow to ensure that the waitlist does not return. The backlog reduction strategy may include: auditing the waitlist to identify whether all people referred still require the service; putting on a finite number of extra clinics; or providing brief consultations by phone or telehealth. It is anticipated that this stage will take between 3 and 6 months. The aim will be to reduce wait time to a target number of weeks, to be determined by the researchers in consultation with key stakeholders from the epilepsy clinic.

Step 4 involves setting up or modifying the neurologists’ booking diaries to match the demand calculation. For example, if the final calculation indicated that 12 new appointments would be required each week to meet average demand, then the 4 neurologists would need an average of 3 new appointment slots available in each weekly clinic.

Step 5 sees a change in process where people referred are booked straight into an appointment without being placed on a waitlist. *All* referrals will be seen promptly and triage decisions regarding priority of ongoing management will be made at the first appointment by the consulting neurologist. Making these changes will likely have a flow-on effect and adjustments to caseload management are anticipated. For example, absorbing a constant flow of new assessments may instigate a review of discharge practices and management of review appointments.

### Outcome measures

Service and patient data will be collected for all people admitted during the trial period from health service databases.

The primary outcome measure will be wait time, measured by (i) the mean number of days waited from referral to first appointment for patients seen across the entirety of the pre and post intervention periods and for each month of the trial and (ii) the number of patients on the wait list at monthly time points.

Secondary outcomes will be service measures, patient outcomes and qualitative data to evaluate staff perceptions (Table 1). Service measures will be rate of failure to attend, rate of discharge, and appointment outcome (attended and scheduled for review; attended and discharged; or failed to attend). In addition, the waiting time of the longest waiters (90th percentile and mean of top 3 waiters) will be collected monthly from the beginning of the implementation and throughout the post-implementation phase. Outcomes from the initial waitlist reduction strategy will be recorded as well as costs associated with implementation. The number of new referrals received will be tracked monthly to monitor changes in demand and provide context for interpretation of results.

**Table 1:** Outcome measures and collection phase

	Pre	During	Post
Primary outcome			
Wait time			
Number of patients on the wait list	✓	✓	✓
Number of days waiting from referral to first appointment	✓		✓
Secondary outcomes			
Service measures			
Number of appointments delivered (new and review)	✓		✓
Outcome (booked for review, failed to attend, discharged)	✓		✓
Wait time to next first appointment and next review appointment		✓	✓
Number of days waited by the longest waiters (90th percentile and top 3)	✓	✓	✓
Outcomes from initial waitlist reduction strategy (booked for assessment or removed from waitlist)		✓	
Cost directly associated with implementation		✓	
Patient outcomes			
Hospital admissions (number and total days)	✓		✓
Emergency department presentations between referral and first appointment	✓		✓
Change in management or risk between referral and first appointment	✓		✓
Staff perception			
Staff focus group			✓

Patient outcomes for the pre and post-intervention groups will include hospital admissions (number and total admitted days) and presentations to Emergency Department between date of referral and first appointment, obtained from routinely collected hospital data. Changes to patient management that occur at the first appointment, such as changes in restrictions to driving or employment or alterations in medication, will also be collected by auditing of a random sample of 100 patient medical files (50 pre and 50 post- intervention) to provide a marker of the impact of delays in accessing care on patient care.

Staff perceptions and their experience of implementing the STAT model will be sought in a focus group that will cover topics such as: how the waitlist was reduced, what support was required to implement STAT, whether there were unexpected consequences, and whether the intervention could be replicated (Table 2). Additional qualitative data will be collected via a research activity log, detailing challenges and facilitators observed. Qualitative and quantitative outcome measures will be combined in the process evaluation.

**Table 2:** Schedule for focus group

Topic Area	Sample Questions
• Context question and general thoughts	• Please describe the changes that were made to your service in relation to the STAT model
• Pre-intervention	• Prior to the change did you perceive waiting times to be a problem in this service, and what are your thoughts on whether there was a case for change?
• Implementation period	• How would you describe your experience during the backlog reduction and setting up the new work processes? • What worked well? • What were the barriers to implementing this change? • What support did you require to implement this model?
• Effect on work practices	• How does the model affect your workload? • Describe changes to the way you manage your patients for ongoing treatment? • Were there any unexpected pathways or consequences that occurred as a result of this change?
• Effect on patient care	• What effect has the model had on patient care?
• Overall opinion/ future directions	• Can you describe any other benefits of the new system? • Can you describe any disadvantages? • Are there things that could have been done differently to improve the implementation process? • Explain your thoughts on whether this model could be implemented in other services • Do you have any other thoughts about this model and its applicability to other services?

Focus group purpose and framework:

• A focus group will be conducted with staff involved in the intervention. The purpose of the focus group is to gain insights into the process of implementing the STAT model from the service provider perspective. Of interest are the group’s views on how the STAT model impacted their workflow, whether there were benefits or drawbacks, what support was required, were there unexpected consequences and whether the model should be continued in this clinic and/or applied to other clinics

Initial statements:

• Let the staff members know that they will be asked questions about their experience of the STAT model

• Remind them that their answers will be recorded so that the researchers can review them later

### Data analysis

Primary and secondary data from the pre and post-intervention cohorts will be compared using *t*-tests for continuous data (waiting time, number of appointments, scheduled appointments, missed appointments, emergency department presentation and hospital admission data, and changes in patient risk and/or management) or equivalent non-parametric tests as appropriate. Chi squared statistics will be used to compare nominal patient-level data for the two groups.

**Fig. 2:**
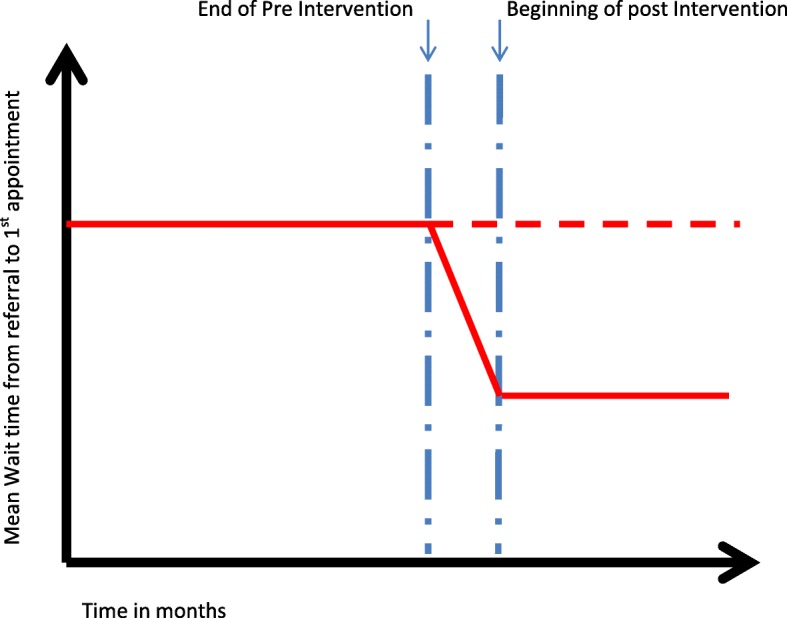
Interrupted time series regression - Hypothesised level change

The mean number of days waited each month and number of people on the waiting list at sequential time points will be analysed using interrupted time series regression, following methods described by Bernal [23]. It is hypothesized that the intervention will show a 40% reduction in wait time and the impact model will be a level change (Fig. 2).

Focus group data will be audio-recorded and transcribed verbatim. Analysis will follow an interpretive description approach [24–26]. Two researchers will independently analyse transcripts line by line and assign codes to text. Researchers will discuss codes and identify themes, and member checking will be conducted to ensure that researchers have correctly interpreted discussion.

**Fig. 3:**
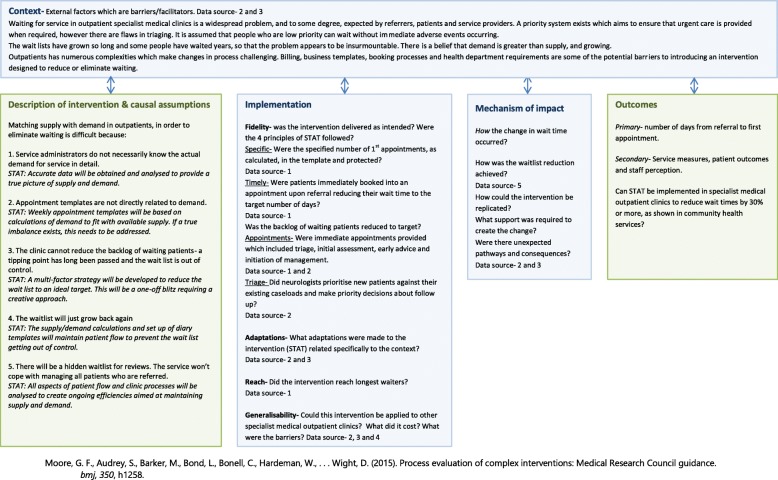
Process Evaluation. Data sources-1-Organisation’s data report, 2-Focus group with staff, 3-Research log, 4-Financial report, 5-Record of backlog reduction actions. Blue boxes are key components of process evaluation. Green boxes indicate intervention and outcomes

Qualitative and quantitative data will be combined and organized for analysis consistent with recommendations for process evaluation of complex interventions [21] (Fig. 3). To evaluate the process, causal assumptions will be described as well as contextual factors that facilitate, or hinder implementation and adaptations made to accommodate the setting. Fidelity will be examined by assessing whether the intervention was implemented according to the principles of the STAT model, such as whether the calculated number of assessment and review appointments were booked in the implementation phase. Reach will be determined by calculating the impact on the lowest priority referrals and those who have waited the longest. Hypotheses about the mechanism of impact, or *how* the change in wait time occurred will be generated in the light of the qualitative and quantitative data.

A sample size of *n* = 975 over the 18-month data collection period will be sufficient to detect a hypothesised reduction in waiting time of 40% with a power of over 0.99, with alpha set to 0.05, based on comparison of mean waiting time pre and post intervention using a two-tailed t-test for independent groups. This estimation is based on historical data for the participating clinic with a pre-intervention mean waiting time of 72 days (SD 85 days). The calculation also assumes a 50% reduction in variability in waiting time data, based on the results of previous STAT trials [6]. There is some uncertainty in power calculations for time series analysis [23, 27], but the proposed number of monthly observations (up to *n* = 24) is expected to be sufficient to provide moderate power given a strong intervention effect, minimal seasonal effects and even distribution of data points are expected [23, 27, 28].

### Discussion

This study will add to the evidence about reducing wait times by finding out if the STAT model of access and triage, previously shown to reduce wait times by 34% in community outpatients [6], can deliver similar results in a specialist medical outpatient clinic. This setting presents new challenges, including longer waiting lists, different funding structures and complex systems for bookings, billing and reporting. However, like its counterparts in community outpatients, this service has experienced relatively stable demand despite the existence of a large waiting list and it uses a combination of assessment and follow-up appointments creating options for flexibility in service delivery. These factors provide reason to believe that implementation of the STAT model could lead to sustainable reductions in waiting time for people seeking care from this epilepsy clinic.

A limitation of the study includes the single group, pre post design, which makes it difficult to control for confounding factors such as policy and staffing changes that could affect wait time during the study period. As a service level intervention, a cluster randomised controlled trial would be the ideal study design to provide rigorous evidence of effectiveness of the STAT model in outpatient clinics. However, given that there are questions over feasibility in this setting, a pre post study with a process evaluation will provide important insights into whether the STAT model can be applied in this setting before progressing to a more complex trial. The use of time-series analysis somewhat mitigates the limitations of a pre post design and can be seen as a strength of the study. The process evaluation is included to enhance interpretation of results and research translation, providing practical and pragmatic information to others seeking to replicate and potentially scale up the intervention.

If this study is found to be effective for reducing wait time and improving equitable access to the epilepsy clinic, then implementation of the STAT model may be applicable to other specialist medical outpatient clinics, and may have significant effects on patient outcomes [29]. At the health service where this study will be completed, a readily available report through the internal database indicates that the number of people waiting for all outpatient services is 37,810. This has additional implications in that outpatient waiting lists have also been described as hidden waiting lists for surgery [30]. More efficient, equitable and accessible delivery of outpatient services has the potential to affect thousands of people, improve health outcomes, prevent adverse events, and enhance patient flow across the health continuum.

## Data Availability

Non-identifiable data for the key outcomes will be available from the authors on request at the conclusion of the study.

## Abbreviation

STAT: Specific Timely Appointments for Triage
